# Human embryonic stem cells: Distinct molecular personalities and applications in regenerative medicine

**DOI:** 10.1002/ca.23318

**Published:** 2019-01-07

**Authors:** Graham Dupont, Emre Yilmaz, Marios Loukas, Veronica Macchi, Raffaele De Caro, R. Shane Tubbs

**Affiliations:** ^1^ Seattle Science Foundation Seattle Washington; ^2^ Department of Anatomical Sciences St. George's University Grenada West Indies; ^3^ Department of Neuroscience Anatomy Institute, University of Padova Padova Italy

**Keywords:** human embryonic stem cells, pluripotency, OCT4, stem cell biology, treatment, molecular, disease, regeneration

## Abstract

The field of stem cell biology is exciting because it provides researchers and clinicians with seemingly unlimited applications for treating many human diseases. Stem cells are a renewable source of pluripotent cells that can differentiate into nearly all human cell types. In this article we focus particularly on human embryonic stem (hES) cells, derived from the inner cell mass of the blastocyst and cultured for expansion while remaining undifferentiated, to explore their unique molecular personalities and clinical applications. The aim of this literature review is to reflect the interest in hES cells and to provide a resource for researchers and clinicians interested in the molecular characteristics of such cells. Clin. Anat. 32:354–360, 2019. © 2018 The Authors. *Clinical Anatomy* published by Wiley Periodicals, Inc. on behalf of American Association of Clinical Anatomists.

## INTRODUCTION

The evolution of biological knowledge, and the use of human embryonic stem (hES) cells following the first report of their derivation from mouse blastocysts in 1981, has given birth to an exciting field, motivating further study of human cell development and differentiation. The level of interest in this field is seldom seen elsewhere (Evans and Kaufman, 1981; Martin, 1981), but the attention hES cells receive is well deserved. Their inherent biological traits (i.e., indefinite proliferation, pluripotency, and genotypic normalcy) have enabled us to envisage boundless applications to disease treatment, cell therapy, and regenerative medicine (Nakatsuji and Suemori, 2002; Fritsch and Singer, 2008). Embryonic stem cells are selected at specific developmental milestones, obtained before endometrial implantation of the blastocyst, the interval before they are embedded in the thickened stratum of uterine wall; they are called “blastomeres” during the blastocyst stage of embryonic cleavage (Standring, 2016). On the fifth day of development, the first differentiation is observed wherein the inner cell mass (ICM) emerges; cells of the ICM are pluripotent (can differentiate into any type of cell) and once harvested can undergo mitosis indefinitely in culture (Cruz et al., 2012; Yu and Thomson, 2016). After they are removed from the blastocyst, ICM cells destined for culture are named hES cells.

The isolation of hES cells led to a more convenient and reliable model for studying human cellular development and differentiation than embryonal carcinoma (EC) cells. At one point, before the work of Thomson, Martin, and Evans with mouse, primate, and human ES cells (Thomson et al., 1995; Pera et al., 2000), EC cells were the gold standard stem cell model. Currently, hES are used in various types of tissue engineering and are undergoing rigorous investigation for their regenerative potential in the treatment of diseases such as Alzheimer's, multiple sclerosis (MS), amyotrophic lateral sclerosis (ALS), cardiovascular ischemic lesions, and diabetes mellitus; this list is not exhaustive (Orlic, 2003; Urbanek et al., 2005; Dimos et al., 2008; Wong and Chang, 2009; Martino et al., 2010; Uccelli et al., 2011; Tabansky and Stern, 2016; Dang et al., 2018).

Considering the growing interest in this field and its promise in disease treatment, regenerative medicine, cell and gene therapy, and in deepening our understanding of human cellular development, detailed discussion of these areas seems appropriate. We also consider the future trajectory of their clinical applications.

## DISCUSSION

### What is an hES Cell?

James Thomson, considered the father of hES cell research and predictor of future discoveries in the field, describes this class of embryonic cells as distinguished by their “developmental plasticity” and by how they can be derived in the first place. hES cells are derived from in vitro‐fertilized embryos, harvested once the ICM sprouts from the inner layer of the blastocyst. The cells of the embryo, although primitive, demonstrate self‐regulatory behavior. An embryo at the pre‐implantation stage, if cut in half, can form two distinct embryos that develop to full term; if two embryos at different developmental milestones are fused, the resulting single aggregate embryo again develops normally and contains genetic information from four different parents (Thomson, 2001). This developmental plasticity is what allows these embryonic cells to mix at different stages of development while still maintaining their ability to develop normally. hES cells are effectively an empty canvas. Once removed from the blastocyst and induced by specific molecular factors, they begin to multiply and differentiate into their respective cellular groupings. The cells of the ICM can differentiate into any cell of the human body, but have limited ability to replenish themselves in utero. Their pluripotent behavior is maintained in cell culture but here they can reproduce and replace themselves indefinitely.

Before hES cells were developed, this phenomenon was practically unheard of in human cells. Teratoma masses contain cells of all three germ layers, but cells isolated from the human ICM were not considered until the work of Thomson in 1998 (Thomson et al., 1998). EC cells were the best models for studying developmental mechanisms and differentiation, as they were the first self‐derived pluripotent cells to be described before ES cells (Kleinsmith and Pierce, 1964). Since they were the stem cells of malignant teratocarcinomas they would metastasize and kill the host when implanted into an adult, though they expressed cells of the three germ layers in vivo. This was one of many drawbacks of EC cells until it was discovered that they could follow a normal developmental cycle if injected into the blastocyst (Gardner, 2013). They could integrate into the blastocyst and give rise to most tissues of the body, but they tended to form teratocarcinomas in chimeric murine offspring at birth or during later development; or those mutations would virtually arrest the embryonic developmental process and kill the fetus before birth (Gardner, 2013). ES cells, derived from the blastocyst instead of testicular tumors, share the pluripotency of EC cells and form teratocarcinomas in syngeneic or immunocompromized non‐syngeneic murine models; otherwise, they solve problem of failure of embryonic development when transplanted into embryonic environments, and address the many other drawbacks of EC cells (Gardner, 2013). ES cells then became the next focus of interest, each discovery setting the stage for the eventual isolation of hES cells.

The excitement about hES cells was unbounded until an ethical firestorm, unleashed by religious and political sectors, ended in defunding and strict limitations being placed on the study of then‐current cell lines; the Bush Administration authorized that only currently existing hES cell lines could be investigated, and no derivation of additional cell lines would be permitted. Regulations have since become more lenient. The embryos for generating hES cell lines are left over from in vitro fertilization or obtained from couples specifically requesting the embryos to be donated to science.

### HES Cell Standards

Discussion of the salient biological minutiae of hES cells must be based on the criteria that provide an inclusive definition of those cells:

- hES cells are derived from the ICM of the human blastocyst at either the pre‐implantation or peri‐implantation stage of embryonic development (Yu and Thomson, 2016);
- hES cells are karyotypically normal even after extensive proliferation; there is no aneuploidy even after several multiplications of the original population (Amit et al., 2000; Kiessling et al., 2003);
- Upon induction, hES cells should differentiate into any of the three embryonic germ layers at any point in the cell culture timeline (Itskovitz‐Eldor et al., 2000; Reubinoff et al., 2000);
- There should be large‐scale cloning with completely suppressed differentiation (Reubinoff et al., 2000);
- hES cells must express the molecular markers of pluripotent cells (see Table 1).

**Table 1:** hES Cell Markers and Genes

Marker	Function	Reference
OCT4	Maintenance of pluripotency, coordinated control of transcriptional, and post‐transcriptional machinery of ICM cells with SOX2	Wang et al. (2012)
SOX2	Maintenance of pluripotency, coordinated control for transcriptional, and post‐ transcriptional machinery of ICM cells with OCT4	Wang et al. (2012)
NANOG	Maintenance of pluripotent gene products, formation of binary transcription complex with OCT4	Wang et al. (2012)
BMP4	Maintenance of pluripotency, self‐renewal, and cellular specification via coordination with OCT4 and SOX2	Loh et al. (2006)
GDF3	Maintenance of self‐renewal via inhibition of p38 MAPK and ERK pathways	Qi et al. (2004)
REX1	Inhibition of BMP, maintenance of pluripotency and undifferentiated cellular state	Levine et al. (2006)
ESG1	Maintenance of pluripotent genes and products	Cowan et al. (2005)
DPPA2	RNA‐binding protein abundantly expressed in the ICM, parallel expression with OCT4	Tanaka et al. (2006)
DPPA4	Maintenance of pluripotent genes and products	Du et al. (2010)
hTERT	Maintenance of functional telomerase activity and proliferative expansion of hES cells	Xu et al. (2004)
TRA1‐60/81	hES cell antigens carried by podocalyxin (200 kDa) membrane protein	Schopperle et al. (2007)

Only a few years after the first hES cells were isolated, Pera suggested that pluripotent ES cells should be able to differentiate spontaneously into any of the three embryonic germ layers. However, this phenomenon had previously been observed only in mouse ES and embryonic germ (EG) cells (Pera et al., 2000). Spontaneous differentiation of ES cells had been well demonstrated in lower vertebrate models, but the capacity of hES cells to transform spontaneously into the three germ layers was not established until much later (Evans and Kaufman, 1981; Martin, 1981; Handyside et al., 1987; Thomson et al., 1995; Itskovitz‐Eldor et al., 2000). Trounson described the maintenance of normal hES cell morphology and karyotype after plating HES‐1‐ and HES‐2‐derived cells on feeder mouse fibroblasts (Reubinoff et al., 2000). No karyotypic mutations were observed after 384 and 264 population doublings, respectively; grafting these ES cells into severe combined immunodeficiency (SCID) mice produced various teratomas in the testicular strata (which had lacked malignant teratocarcinomas) containing tissues from all three germ layers such as cartilage, squamous epithelium, primitive neuroectoderm, neuronal superstructures, muscle, bone, and glandular epithelia (Reubinoff et al., 2000). In addition to reversing disease, hES cells can infiltrate and replenish damaged regions of organs after implantation, for example, remuscularization of infarcted myocardium, neuronal precursor cells for repairing CNS injury, and regeneration of glucose‐responsive pancreatic tissue (Zhang et al., 2001; Laflamme et al., 2007; Kroon et al., 2008).

### Molecular Anatomy and Physiological Characteristics of hES Cells

As mentioned above, the molecular markers and surface antigens of pluripotent cells are among the defining criteria for hES cells. Early in the history of stem cell research, those markers allowed scientists to confirm that ES and EC cells were analogous in respect of their molecular personalities, and they now enable us to monitor the differentiation and developmental behavior of these cells in vitro and in vivo.

Among the various markers of hES cells (see Table 1) unique to early embryonic cell maintenance, communication, and proliferation is OCT4 (also seen as OCT3, OCT3/4), a major directing transcription factor with particular expression in early embryonic cells, encoded by the *Pou5f1* gene and frequently used as a marker for pluripotency (Shamblott and Sterneckert, 2004; Shi and Jin, 2010; Zeineddine et al., 2014). OCT4 maintains the ICM while preventing the differentiation of this mass of cells into trophectoderm (Nichols et al., 1998). Knocking out OCT4 prevents formation of the ICM. When it is absent, cells destined to form the ICM differentiate into members of the extraembryonic trophoblast lineage, and proliferation of the trophoblast is restricted (Nichols et al., 1998). Fibroblast growth factor‐4 (FGF4), a protein activated by OCT4 expression, restores the proliferative potential of the trophoblast cells (Tanaka et al., 1998). OCT4 expression surges in pluripotent cells, preventing them from transforming from their undifferentiated state. OCT4 can also induce somatic cells to pluripotency, a technique now used for preparing iPS cells (Shi and Jin, 2010; Zhu et al., 2010). Acting together with OCT4 are SOX2 and NANOG, transcription factors that suppress the specification of pluripotent cells and maintain their capacity for self‐renewal (Wang et al., 2012). OCT4 and SOX2 operate in tandem and form a complex at the sox‐oct element of *Sox2*. As further evidence for their roles in suppressing differentiation, polarized expression, or knockout of OCT4, SOX2, or NANOG leads to lineage specification of ICM cells (Wang et al., 2012). NANOG is crucial in this process as it monitors and maintains the expression of OCT4 and SOX2 by interacting with their respective genes, *Pou5f1* and *Sox2*. Furthermore, through regulation of *Fox3d* and *Setdb1*, NANOG exerts control over cellular fate determination (Loh et al., 2006). BMP4 also assists in maintaining pluripotency and ES cell self‐renewal via inhibition of the extracellular receptor kinase (ERK) and p38 mitogen‐activated protein kinase (MAPK) pathways, responsible for downstream signaling of mitogens and growth factors that induce cellular division and differentiation, for example, LIF, FGF, and BMP (Qi et al., 2004). Qi et al. demonstrated that introduction of exogenous BMP4 to BMP4‐null ES cells causes an immediate reduction in activity of both ERK and MAPK (Qi et al., 2004). Members of the transforming growth beta (TGFB) pathway, LEFTY1, LEFTY2, and GDF3, are also expressed in pluripotent cells, declining sharply after cellular fate designation (Levine and Brivanlou, 2006). Other important markers of hES cells include REX1 (Cowan et al., 2005), ESG1 (Tanaka et al., 2006), DDPA2 (Du et al., 2010), hTERT (Xu et al., 2004), TRA‐1‐60, and TRA‐1‐81 (Schopperle and DeWolf, 2007) (see Table 1).

### Markers of Induced Progenitor Cells

To specify a particular cell lineage, hES cells must be bathed in molecular factors that designate them for the desired cellular fate. Brachyury, a member of the T‐box family of genes, is an essential transcription factor that allows the developmental environment, or niche, for sustained growth and differentiation of mesodermal cells to be accessed (Keller et al., 1993; Martin and Kimelman, 2010). Zeta‐globin, a common marker for immature hematopoetic stem cells, has also been used to induce pluripotent stem cells into the mesodermal lineage (Itskovitz‐Eldor et al., 2000). The erythyroid‐specific transcription factor NF‐E1 also demonstrates coordinated expression with the globins for specification and growth of hematopoietic cells (Lindenbaum and Grosveld, 1990). Adipose cells, also of mesodermal origin, can be induced via retinoic acid (RA) with dimethyl‐sulfoxide (DMSO), yielding high levels of adipogenesis. The hES‐derived adipocytes typically express glycerol‐3‐phosphate dehydrogenase (GPDH) (a necessary enzyme for fat metabolism) and adipocyte‐lipid binding protein (ALBP). Dani et al. induced the ZIN40, E14TG2a, and CGR8 stem cell lines into adipocytes using RA and an adipogenic hormone medium (insulin and triiodothyronine), and found these lines to contain fully differentiated adipocytes, as indicated by observations of triglyceride metabolism in the induced cells (Dani et al., 1997). Schuldiner et al. (2000) determined through identification of various growth factors that Activin‐A and TGFβF1 also contribute to the induction of mesodermal cells, and RA, epidermal growth factor (EGF), BMP‐4, and FGF induce mesodermal and ectodermal specification (Schuldiner et al., 2000). It was further determined that nerve growth factor (NGF) and hepatocyte growth factor (HGF) can induce specification into any of the three embryonic germ layers (Schuldiner et al., 2000).

Cardiomyocytes, readily identified by α‐smooth muscle actin and β‐myosin expression (Laflamme et al., 2007; Leor et al., 2007), have been derived from hES cells (71%–95% purity) using a BMP‐4/Activin‐A system. Their transplantation into infarcted cardiac tissue offers promising, non‐invasive alternatives to placement of pacemakers. However, when there is extensive tissue death in the myocardium of the left ventricle, for example, calculated measures must be taken to ensure delivery of a sufficient number of pure cardiomyocytes to the infarcted area, which relates directly to the development of methods for producing and converting hES cells on a large scale. Interestingly, Laflamme et al. demonstrated a 90% engraftment success rate using cardiomyocytes derived from hES cells into uninjured murine myocardium, with full functionality and electromechanical coupling to the heart's conduction system (Laflamme et al., 2005), but the graft rate when the cells were inserted into infarcted tissue was only 18%, suggesting some ischemia‐related or inflammatory mechanism for rejecting the transplanted cardiomyocytes (Laflamme et al., 2007). A “survivability cocktail” was designed to address this problem, consisting of Matrigel to prevent anoikis, peptide Bcl‐XL and cyclosporin‐A to mitigate mitochondria‐directed cell death, pinacidil to influence ATP‐gated K^+^ channels, IGF‐1, and a caspase inhibitor to enhance metabolic functionality; when these were combined there was a sevenfold increase in graft area (Laflamme et al., 2007). Multifold processes of cell death after transplantation might be navigated by exploring methods of survivability, requiring an extensive understanding of the molecular basis of rejection, which currently poses a challenge in regenerative medicine.

Cells of the nervous system demonstrate elegant variation, each with unique molecular characteristics; this is also true of early neuronal cells. SOX1 is generally the first marker expressed once the hES cell commits to the neural lineage, and then through its neuroepithelial expansion, PAX6 is observed. Downstream, neural cells committed to the anterior CNS are marked by FOXG1, and those of the neural crest by p75 (see Table 2) (Chambers et al., 2009). The therapeutic potential of hES‐derived midbrain dopamine neurons is also being explored as a route to reversing the effects of Parkinson's disease, the development of which results in the death of dopaminergenic neurons, leading to the characteristic tremors in patients with this disease (Perrier et al., 2004). Noggin (NOG), an effective BPM antagonist and inhibitor of downstream TGFβ growth factor signaling, is widely used to induce hES cells to the neuronal lineage with high yield and functionality (Lim et al., 2000). Furthermore, co‐induction with SB431542, an inhibitor of growth factors LEFTY, Activin, and TGFβ, makes the efficiency much greater (over 80% induction) than the use of NOG or SB431542 alone (<10%) (Chambers et al., 2009). hES cells have been used to generate retinal precursor cells, known to be scarce or even absent in human adults. Successful derivation of RPE cells uses BMP signaling antagonists and the inductive factor for designation to the neural retinal cell lineage, insulin‐like growth factor‐1 (IGF‐1) (Lamba et al., 2006). The expression of transcription factors for early eye field development such as PAX6, abundant during retinal cell development but not expressed when the cell progresses to full maturity, and MITF and OX2, is also necessary for growth and functionality. The Wnt/beta‐catenin pathway also influences the differentiation of retinal pigmented epithelial (RPE) cells through regulation of MITF and OX2 (Fujimura et al., 2009; Westenskow et al., 2009).

**Table 2:** Various Cells Induced From hES Cells and Their Molecular Markers

Cell Type	Marker		Reference
Endodermal	α‐Fetoprotein		Itskovitz‐Eldor et al. (2000)
Mesodermal	ζ‐Globin		Itskovitz‐Eldor et al. (2004); Tanaka et al. (1998, 2006)
	Brachyury		
Ectodermal	Neurofilament 68 kD		Itskovitz‐Eldor et al. (2000)
Cardiomyocytes	β‐Myosin		Laflamme et al. (2007); Keller et al. (1993)
	α‐Smooth muscle actin		
Adipocytes	Glyceraldehyde‐phosphate‐dehydrogenase(GPHD)		Xu et al. (2004)
	Adipocyte lipid binding protein (ALBP)		
	Adipsin		
	Peroxisome proliferative‐activated receptor (PPAR)		
Hematopoetic	βHI		Du et al. (2010)
	β major globin		
	GATA‐1		
	GATA‐3		
Neuronal tissue	β‐III tubulin (immature/mature axons)	PAX6 (neuroepithelial)	Dani et al. (1997); Ford (1998)
	ZIC1 (neuroepithelial)	p75 (neural crest)	
	FOXG1 (anterior CNS)	NF70 (immature axons)	
	SMI32 (immature axons)		
Pancreatic β‐cells	GLUT2		Lim et al. (2000)
	Islet‐specific GK		

### Future Trajectory for hES Cells in Regenerative Medicine

The promise of human embryonic stem cells is that one day we will be able to generate any cell type so that the effects of many injuries, diseases, cancers, and degenerative disorders can be completely reversed. However, there are many academic and institutional barriers to the fulfillment of that promise: identifying and controlling the specific molecular pathways that designate the lineage of a cell type or demonstrating their functionality in vivo and pushing their use to preclinical and clinical paradigms. Countless cell types derived from hES cells can demonstrate full functionality and are aimed at reversing major diseases such as diabetes, Alzheimer's, Parkinson's, ALS, and MS, and macular degeneration. They even promise to reverse many types of paralysis from spinal cord injury.

Derivation of insulin‐ and glucose‐responsive cells from hES cells has been well demonstrated, and other peptide hormone‐secreting cells able to generate somatostatin, pancreatic polypeptide, and ghrelin have been successfully differentiated (Assady et al., 2001; Kroon et al., 2008). Assady et al. (2001) isolated insulin‐secreting pancreatic β‐cells in high yield and showed them to express the genes for GLUT2 and islet‐specific GK, vital for the glucose‐responsive secretion of insulin. Though these cells have been successfully derived in vitro with hormonal functionality and sensitivity, imitating true embryonic conditions remains a challenge. Solving one of the problems that concerns the production of many highly specialized cells, directing their stepwise differentiation, requires precise developmental models and conditions. D'Amour et al. directed the precise development of insulin‐secreting cells through a five stage differentiation model, from OCT4 and NANOG expression as endodermal cells, to their expansion into primitive gut tube, posterior foregut, pancreatic endoderm and endocrine precursor, and finally hormone‐expressing pancreatic cells (D'Amour et al., 2006). Fully functional pancreatic β‐cells derived from hES cells would effectively replace exogenous insulin treatment as a renewable source of cellular therapy, greatly enhancing the quality of life for countless individuals affected by type 1 diabetes.

Parkinson's disease involves the progressive death of dopaminergenic neurons in the substantia nigra of the midbrain, resulting in neuromuscular symptoms such as tremors, rigidity, and retardation of movement. Furthermore, many individuals with Parkinson's disease (upwards of 40% affected) report a myriad of different types of musculoskeletal or neuropathic pain, exacerbated by the constant hypertonicity of skeletal muscle and neuronal death, ranging from aching and cramping to dystonia in the face and pharynx, along with burning sensations in the skin (Ford, 1998). Dopaminergic neurons of the midbrain function by raising the pain threshold to conscious perception, and their dysfunction and further death as a result of progressing Parkinson's could be reversible via transplantation of dopaminergic neurons derived from hES cells. Dopaminergenic neurons have been obtained successfully in vivo (Park et al., 2005), and full functionality and high levels of dopamine release (1,283 ± 421 pg/mL) were reported by Perrier et al. (2004).

Spinal cord injury is another serious clinical issue with few options for treatment, only management through medication, myoelectrically controlled neuroprostheses to restore hand and some lower extremity function (Ho et al., 2014), physical and occupational therapy, and supportive consultation. These options are not known to restore full function, but only partial motion of certain areas using myoelectrical prosthetics. The transplantation of hES cell‐derived oligodendrocyte progenitor cells is an exciting possibility for preclinical models of spinal cord injury treatment, aimed at rejuvenation through remyelination of the affected areas. Keirstead et al. (2005) reported successful and functionally‐directed differentiation of hES cells into oligodendrocyte progenitor cells that could remyelinate and improve locomotion in adult rats with spinal cord injury. Neuroprogenitor cells have also been grafted in primates with spinal cord injury by Rosenzweig et al. (2018); robust axonal regeneration with fully functional synapses expanded outward from the engraftment area and formed a neural relay site with existing spinal cord tissue. Additionally, corticospinal axons, known for their role in voluntary skeletal muscle contraction, migrated into the graft site in these primates, with enhanced limb functionality (Rosenzweig et al., 2018). These findings point toward future achievement of the same results in the clinic, but regenerative medicine and stem cell research still face significant barriers: establishing the extensive biological and molecular knowledge necessary for directed differentiation in vivo, and the prevention of cell death after transplantation.

## CONCLUSION

Stem cell research remains a frontier of research with potential for achievements beyond the imagination. From restoring insulin function in diabetics to restoring locomotion in individuals affected by paralysis, each new discovery brings us closer to treating even the most intractable diseases so that individuals can live in health, unencumbered by sickness.

## References

[ca23318-bib-0001] Amit M , Carpenter MK , Inokuma MS , Chiu CP , Harris CP , Waknitz MA , Itskovitz‐Eldor J , Thomson JA . 2000 Clonally derived human embryonic stem cell lines maintain pluripotency and proliferative potential for prolonged periods of culture. Dev Biol 227:271–278. 11071754 10.1006/dbio.2000.9912

[ca23318-bib-0002] Assady A , Maor G , Itskovitz‐Eldor J . 2001 Insulin production by human embryonic stem cells. Diabetes 50:1691–1697. 11473026 10.2337/diabetes.50.8.1691

[ca23318-bib-0003] Chambers S , Fasano C , Papapetrou E . 2009 Highly efficient neural conversion of human ES and iPS cells by dual inhibition of SMAD signaling. Nat Biotechnol 27:275–280. 19252484 10.1038/nbt.1529 PMC2756723

[ca23318-bib-0004] Cowan CA , Atienza J , Melton DA , Eggan K . 2005 Nuclear reprogramming of somatic cells after fusion with human embryonic stem cells. Science 309:1369–1373. 16123299 10.1126/science.1116447

[ca23318-bib-0005] Cruz M , Garrido N , Herrero J , Pérez‐Cano I , Muñoz M , Meseguer M . 2012 Timing of cell division in human cleavage‐stage embryos is linked with blastocyst formation and quality. Reprod Biomed Online 25:371–381. 22877944 10.1016/j.rbmo.2012.06.017

[ca23318-bib-0006] D'Amour KA , Bang AG , Eliazer S , Kelly OG , Agulnick AD , Smart NG , Moorman MA , Kroon E , Carpenter MK , Baetge EE . 2006 Production of pancreatic hormone‐expressing endocrine cells from human embryonic stem cells. Nat Biotechnol 24:1392–1401. 17053790 10.1038/nbt1259

[ca23318-bib-0007] Dang LT , Phan NK , Truong KD . 2018 Mesenchymal stem cells for diabetes mellitus treatment: new advances. Biomed Res Ther 1:1062–1081.

[ca23318-bib-0008] Dani C , Smith AG , Dessolin S , Leroy P , Staccini L , Villageois P , Darimont C , Ailhaud G . 1997 Differentiation of embryonic stem cells into adipocytes in vitro. J Cell Sci 110:1279–1285. 9202388 10.1242/jcs.110.11.1279

[ca23318-bib-0009] Dimos JT , Rodolfa KT , Niakan KK , Weisenthal LM , Mitsumoto H , Chung W , Croft GF , Saphier G , Leibel R , Goland R , Wichterle H , Henderson CE , Eggan K . 2008 Induced pluripotent stem cells generated from patients with ALS can be differentiated into motor neurons. Science 321:1218–1221. 18669821 10.1126/science.1158799

[ca23318-bib-0010] Du J , Chen T , Zou X , Xiong B , Lu G . 2010 Dppa knockdown‐induced differentiation and repressed proliferation of mouse embryonic stem cells. J Biochem 147:265–271. 19846433 10.1093/jb/mvp161

[ca23318-bib-0011] Evans MJ , Kaufman MH . 1981 Establishment in culture of pluripotential cells from mouse embryos. Nature 292:154–156. 7242681 10.1038/292154a0

[ca23318-bib-0012] Ford B . 1998 Pain in Parkinson's disease. Clin Neurosci 5:63–72. 10785830

[ca23318-bib-0013] Fritsch MK , Singer DB . 2008 Embryonic stem cell biology. Adv Pediatr 55:43–77. 19048727 10.1016/j.yapd.2008.07.006

[ca23318-bib-0014] Fujimura N , Taketo MM , Mori M , Korinek V , Kozmik Z . 2009 Spatial and temporal regulation of Wnt/beta‐catenin signaling is essential for development of the retinal pigment epithelium. Dev Biol 334:31–45. 19596317 10.1016/j.ydbio.2009.07.002

[ca23318-bib-0015] Gardner RL . 2013 Pluripotent stem cells from vertebrate embryos: present perspective and future challenges Essentials of Stem Cell Biology. New York: Academic Press p 23–25.

[ca23318-bib-0016] Handyside A , Hooper ML , Kaufman MH , Wilmut I . 1987 Towards the isolation of embryonal stem cell lines from the sheep. Roux's Arch Dev Biol 196:185–190. 28305842 10.1007/BF00376313

[ca23318-bib-0017] Ho CH , Triolo RJ , Elias AL , Kilgore KL , DiMarco AF , Bogie K , Vette AH , Audu ML , Kobetic R , Chang SR , Chan KM , Dukelow S , Bourbeau DJ , Brose SW , Gustafson KJ , Kiss ZH , Mushahwar VK . 2014 Functional electrical stimulation and spinal cord injury. Phys Med Rehabil Clin N Am 25:631–654. 25064792 10.1016/j.pmr.2014.05.001 PMC4519233

[ca23318-bib-0018] Itskovitz‐Eldor J , Schuldiner M , Karsenti D , Eden A , Yanuka O , Amit M , Soreq H , Benvenisty N . 2000 Differentiation of human embryonic stem cells into embryoid bodies compromising the three embryonic germ layers. Mol Med 6:88–95. 10859025 PMC1949933

[ca23318-bib-0019] Keirstead HS , Nistor G , Bernal G , Totoiu M , Cloutier F , Sharp K , Steward O . 2005 Human embryonic stem cell‐derived oligodendrocyte progenitor cell transplants remyelinate and restore locomotion after spinal cord injury. J Neurosci 25:4694–4705. 15888645 10.1523/JNEUROSCI.0311-05.2005 PMC6724772

[ca23318-bib-0020] Keller G , Kennedy M , Papayannopoulou T , Wiles MV . 1993 Hematopoietic commitment during embryonic stem cell differentiation in culture. Mol Cell Biol 13:473–486. 8417345 10.1128/mcb.13.1.473-486.1993 PMC358927

[ca23318-bib-0021] Kiessling AA , Anderson S , Anderson SC . 2003 Human Embryonic Stem Cells: An Introduction to the Science and Therapeutic Potential. Boston, MA: Jones & Bartlett Learning.

[ca23318-bib-0022] Kleinsmith IJ , Pierce GB . 1964 Multipotentiality of single embryonal carcinoma cells. Cancer Res 24:1544–1551. 14234000

[ca23318-bib-0023] Kroon E , Martinson LA , Kadoya K , Bang AG , Kelly OG , Eliazer S , Young H , Richardson M , Smart NG , Cunningham J , Agulnick AD , D'Amour KA , Carpenter MK , Baetge EE . 2008 Pancreatic endoderm derived from human embryonic stem cells generates glucose‐responsive insulin‐secreting cells in vivo. Nat Biotechnol 26:443–452. 18288110 10.1038/nbt1393

[ca23318-bib-0024] Laflamme MA , Chen KY , Naumova AV , Muskheli V, Fugate JA, Dupras SK, Reinecke H, Xu C, Hassanipour M, Police S, O'Sullivan C, Collins L, Chen Y, Minami E, Gill EA, Ueno S, Yuan C, Gold J, Murry CE. 2007 Cardiomyocytes derived from human embryonic stem cells in pro‐survival factors enhance function of infarcted rat hearts. Nat Biotechnol 25:1015–1024. 17721512 10.1038/nbt1327

[ca23318-bib-0025] Laflamme MA , Gold J , Xu C , Hassanipour M , Rosler E , Police S , Muskheli V , Murry CE . 2005 Formation of human myocardium in the rat heart from human embryonic stem cells. Am J Pathol 167:663–671. 16127147 10.1016/S0002-9440(10)62041-X PMC1698736

[ca23318-bib-0026] Lamba DA , Karl MO , Ware CB , Reh TA . 2006 Efficient generation of retinal progenitor cells from human embryonic stem cells. Proc Natl Acad Sci USA 103:12769–12774. 16908856 10.1073/pnas.0601990103 PMC1568922

[ca23318-bib-0027] Leor J , Gerecht S , Cohen S , Miller L , Holbova R , Ziskind A , Shachar M , Feinberg MS , Guetta E , Itskovitz‐Eldor J . 2007 Human embryonic stem cell transplantation to repair the infarcted myocardium. Heart 93:1278–1284. 17566061 10.1136/hrt.2006.093161 PMC2000918

[ca23318-bib-0028] Levine AJ , Brivanlou AH . 2006 GDF3, a BMP inhibitor, regulates cell fate in stem cells and early embryos. Development 133:209–216. 16339188 10.1242/dev.02192

[ca23318-bib-0029] Lim DA , Tramontin AD , Trevejo JM , Herrera DG , García‐Verdugo JM , Alvarez‐Buylla A . 2000 Noggin antagonizes BMP signaling to create a niche for adult neurogenesis. Neuron 28:713–726. 11163261 10.1016/s0896-6273(00)00148-3

[ca23318-bib-0030] Lindenbaum MH , Grosveld F . 1990 An in vitro globin gene switching model based on differentiated embryonic stem cells. Genes Dev 4:2075–2085. 2269427 10.1101/gad.4.12a.2075

[ca23318-bib-0031] Loh YH , Wu Q , Chew JL , et al. 2006 The Oct 4 and Nanog transcription network regulates pluripotency in mouse embryonic stem cells. Nat Genet 38:431–440. 16518401 10.1038/ng1760

[ca23318-bib-0032] Martin BL , Kimelman D . 2010 Brachyury establishes the embryonic mesodermal progenitor niche. Genes Dev 24:2778–2783. 21159819 10.1101/gad.1962910 PMC3003196

[ca23318-bib-0033] Martin GR . 1981 Isolation of a pluripotent cell line from early mouse embryos cultured in medium conditioned by teratocarcinoma stem cells. Proc Natl Acad Sci USA 78:7634–7638. 6950406 10.1073/pnas.78.12.7634 PMC349323

[ca23318-bib-0034] Martino G , Franklin RJ , Baron Van Evercooren A , Kerr DA , Stem Cells in Multiple Sclerosis (STEMS) Consensus Group . 2010 Stem cell transplantation in multiple sclerosis: current status and future prospects. Nat Rev Neurol 6:247–255. 20404843 10.1038/nrneurol.2010.35

[ca23318-bib-0035] Nakatsuji N , Suemori H . 2002 Embryonic stem cell lines of nonhuman primates. Sci World J 2:1762–1773. 10.1100/tsw.2002.829 PMC6009524 12806169

[ca23318-bib-0036] Nichols J , Zevnik B , Anastassiadis K , Niwa H , Klewe‐Nebenius D , Chambers I , Schöler H , Smith A . 1998 Formation of pluripotent stem cells in the mammalian embryo depends on the POU transcription factor. Cell 95:379–391. 9814708 10.1016/s0092-8674(00)81769-9

[ca23318-bib-0037] Orlic D . 2003 Adult bone marrow stem cells regenerate myocardium in ischemic heart disease. Ann N Y Acad Sci 996:152–157. 12799293 10.1111/j.1749-6632.2003.tb03243.x

[ca23318-bib-0038] Park CH , Minn YK , Lee JY , Choi DH, Chang MY, Shim JW, Ko JY, Koh HC, Kang MJ, Kang JS, Rhie DJ, Lee YS, Son H, Moon SY, Kim KS, Lee SH. 2005 In vitro and in vivo analyses of human embryonic stem cell‐derived dopamine neurons. J Neurochem 92:1265–1276. 15715675 10.1111/j.1471-4159.2004.03006.x

[ca23318-bib-0039] Pera MF , Reubinoff B , Trounson A . 2000 Human embryonic stem cells. J Cell Sci 113:5–10. 10591620 10.1242/jcs.113.1.5

[ca23318-bib-0040] Perrier A , Tabar V , Barberi T . 2004 Derivation of midbrain dopamine neurons from human embryonic stem cells. Proc Natl Acad Sci USA 101:12543–12548. 15310843 10.1073/pnas.0404700101 PMC515094

[ca23318-bib-0041] Qi X , Li TG , Hao J , Hu J , Wang J , Simmons H , Miura S , Mishina Y , Zhao GQ . 2004 BMP4 supports self‐renewal of embryonic stem cells by inhibiting mitogen‐activated protein kinase pathways. Proc Natl Acad Sci USA 101:6027–6032. 15075392 10.1073/pnas.0401367101 PMC395917

[ca23318-bib-0042] Reubinoff BE , Pera MF , Fong CY , Trounson A , Bongso A . 2000 Embryonic stem cell lines from human blastocysts: somatic differentiation in vitro. Nat Biotechnol 18:399–404. 10748519 10.1038/74447

[ca23318-bib-0043] Rosenzweig ES , Brock JH , Lu P , et al. 2018 Restorative effects of human neural stem cell grafts on the primate spinal cord. Nat Med 24:484–490. 29480894 10.1038/nm.4502 PMC5922761

[ca23318-bib-0044] Schopperle WM , DeWolf WC . 2007 The TRA‐1‐60 and TRA‐1‐81 human pluripotent stem cell markers are expressed on podocalyxin in embryonal carcinoma. Stem Cells 25:723–730. 17124010 10.1634/stemcells.2005-0597

[ca23318-bib-0045] Schuldiner M , Yanuka O , Itskovitz‐Eldor J , Melton DA , Benvenisty N . 2000 Effects of eight growth factors on the differentiation of cells derived from human embryonic stem cells. Proc Natl Acad Sci USA 97:11307–11312. 11027332 10.1073/pnas.97.21.11307 PMC17196

[ca23318-bib-0046] Shamblott MJ , Sterneckert JL . 2004 Human Embryonic Stem Cells. New York: Garland Science.

[ca23318-bib-0047] Shi G , Jin Y . 2010 Role of Oct 4 in maintaining and regaining stem cell pluripotency. Stem Cell Res Ther 1:39. 21156086 10.1186/scrt39 PMC3025441

[ca23318-bib-0048] Standring S . 2016 Gray's Anatomy: The Anatomical Basis of Clinical Practice. New York: Elsevier Health Sciences.

[ca23318-bib-0049] Tabansky I , Stern JNH . 2016 Basics of stem cell biology as applied to the brain In: Pfaff D, Christen Y, editors. Stem Cells in Neuroendocrinology. Cham (CH): Springer. 28590705

[ca23318-bib-0050] Tanaka S , Kunath T , Hadjantonakis AK , Nagy A , Rossant J . 1998 Promotion of trophoblast stem cell proliferation by FGF4. Science 282:2072–2075. 9851926 10.1126/science.282.5396.2072

[ca23318-bib-0051] Tanaka TS , Lopez de Silanes I , Sharova LV , Akutsu H , Yoshikawa T , Amano H , Yamanaka S , Gorospe M , Ko MS . 2006 Esg1, expressed exclusively in preimplantation embryos, germline, and embryonic stem cells, is a putative RNA‐binding protein with broad RNA targets. Dev Growth Differ 48:381–390. 16872451 10.1111/j.1440-169X.2006.00875.x

[ca23318-bib-0052] Thomson J . 2001 The Human Embryonic Stem Cell Debate: Science, Ethics, and Public Policy. Cambridge: MIT Press.

[ca23318-bib-0053] Thomson JA , Itskovitz‐Eldor J , Shapiro SS , Waknitz MA , Swiergiel JJ , Marshall VS , Jones JM . 1998 Embryonic stem cell lines derived from human blastocysts. Science 282:1145–1147. 9804556 10.1126/science.282.5391.1145

[ca23318-bib-0054] Thomson JA , Kalishman J , Golos TG , Durning M , Harris CP , Becker RA , Hearn JP . 1995 Isolation of a primate embryonic stem cell line. Proc Natl Acad Sci USA 92:7844–7848. 7544005 10.1073/pnas.92.17.7844 PMC41242

[ca23318-bib-0055] Uccelli A , Laroni A , Freedman MS . 2011 Mesenchymal stem cells for the treatment of multiple sclerosis and other neurological diseases. Lancet Neurol 10:649–656. 21683930 10.1016/S1474-4422(11)70121-1

[ca23318-bib-0056] Urbanek K , Torella D , Sheikh F , De Angelis A, Nurzynska D, Silvestri F, Beltrami CA, Bussani R, Beltrami AP, Quaini F, Bolli R, Leri A, Kajstura J, Anversa P. 2005 Myocardial regeneration by activation of multipotent cardiac stem cells in ischemic heart failure. Proc Natl Acad Sci USA 102:8692–8697. 15932947 10.1073/pnas.0500169102 PMC1150816

[ca23318-bib-0057] Wang Z , Oron E , Nelson B , Razis S , Ivanova N . 2012 Distinct lineage specification roles for NANOG, OCT4, and SOX2 in human embryonic stem cells. Cell Stem Cell 10:440–454. 22482508 10.1016/j.stem.2012.02.016

[ca23318-bib-0058] Westenskow P , Piccolo S , Fuhrmann S . 2009 Beta‐catenin controls differentiation of the retinal pigment epithelium in the mouse optic cup by regulating Mitf and Otx2 expression. Development 136:2505–2510. 19553286 10.1242/dev.032136 PMC2709060

[ca23318-bib-0059] Wong DJ , Chang HY . 2009 Skin tissue engineering. Cambridge: Harvard Stem Cell Institute. 20614591

[ca23318-bib-0060] Xu C , Jiang J , Sottile V , McWhir J , Lebkowski J , Carpenter MK . 2004 Immortalized fibroblast‐like cells derived from human embryonic stem cells support undifferentiated cell growth. Stem Cells 22:972–980. 15536188 10.1634/stemcells.22-6-972

[ca23318-bib-0061] Yu J , Thomson JA . 2016 NIH Stem Cell Information Home Page. National Institutes of Health, U.S. Department of Health and Human Services. URL: https://stemcells.nih.gov/info/Regenerative_Medicine/2006Chapter1.htm [accessed Sept. 2018].

[ca23318-bib-0062] Zeineddine D , Hammoud AA , Mortada M , Boeuf H . 2014 The Oct 4 protein: more than a magic stemness marker. Am J Stem Cells 3:74–82. 25232507 PMC4163606

[ca23318-bib-0063] Zhang SC , Wernig M , Duncan ID , Brüstle O , Thomson JA . 2001 In vitro differentiation of transplantable neural precursors from human embryonic stem cells. Nat Biotechnol 19:1129–1133. 11731781 10.1038/nbt1201-1129

[ca23318-bib-0064] Zhu S , Li W , Zhou H , Wei W , Ambasudhan R , Lin T , Kim J , Zhang K , Ding S . 2010 Reprogramming of human primary somatic cells by OCT4 and chemical compounds. Cell Stem Cell 7:651–655. 21112560 10.1016/j.stem.2010.11.015 PMC3812930

